# Supplementary material for: ATM Pathway Is Essential for HPV–Positive Human Cervical Cancer-Derived Cell Lines Viability and Proliferation
Source: Pathogens. 2022 Jun 1;11(6):637. doi: 10.3390/pathogens11060637 (PMC9228918; doi:10.3390/pathogens11060637)
Supplement: Supplementary file 1 [file pathogens-11-00637-s001.zip › pathogens-1715849-supplementary.pdf]

Abjaude et al., Figure S1

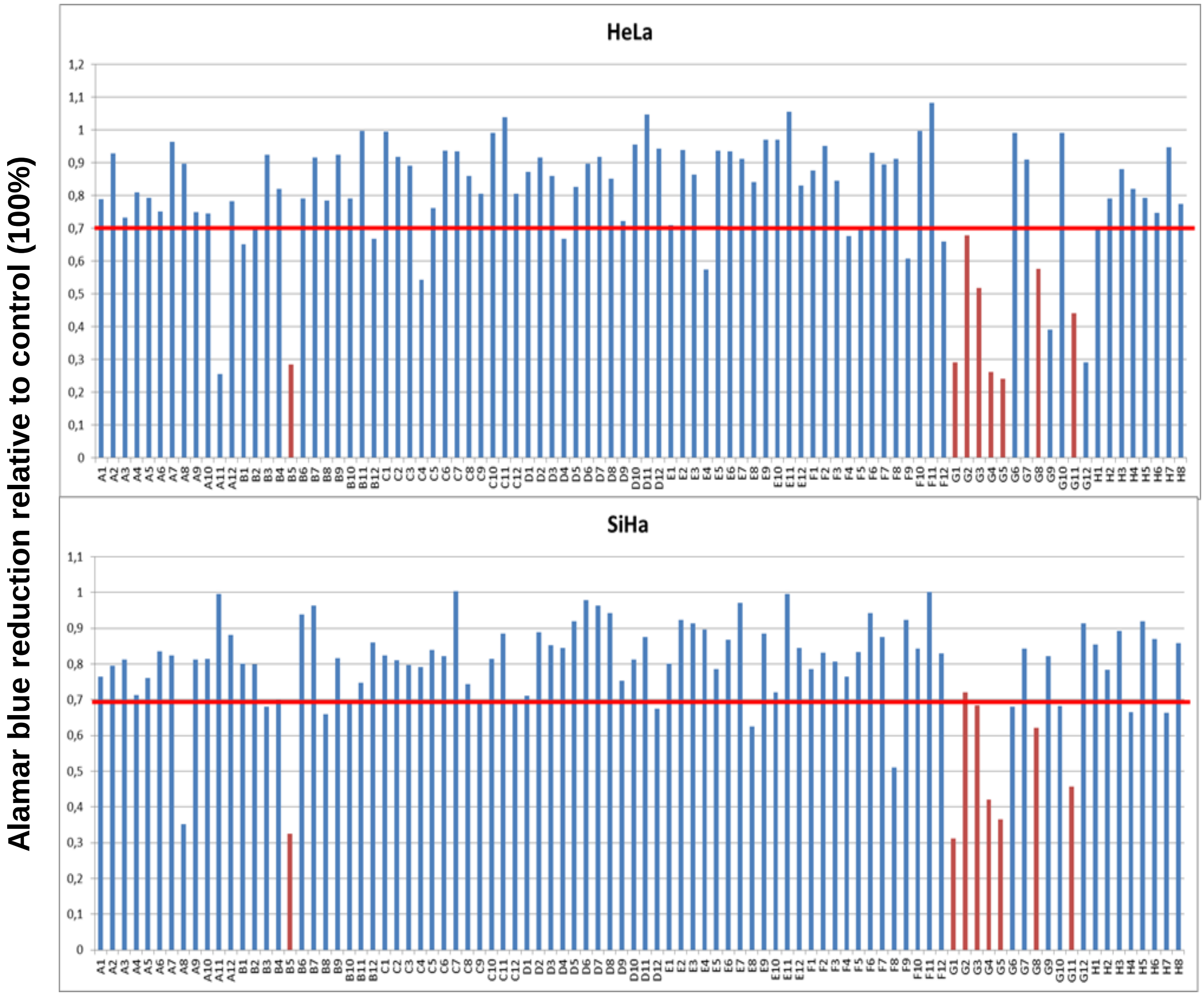

Figure S1– **Screening for genes essential to cervical cancer-derived cell lines viability.** A lentiviral shRNA library (MISSION® DNA Repair Pathway) that targets 116 genes involved in DNA damage signaling/repair was used to silence specific genes in cervical cancer cell lines HeLa and SiHa. Cells were seeded in 96 wells plates (2000 cells/well) and infected with lentiviral particles expressing specific shRNAs after 24 hours. All infections were performed in triplicate. After 72 hours Alamar Blue was added (10 µL/well). After 4 hours Alamar Blue reduction was determined by measuring absorbance at 570 and 600 nm. Viability/proliferation inhibition values are presented as relative to those observed in control cells (cells from the respective type transduced with a scramble shRNA).

Figure S2

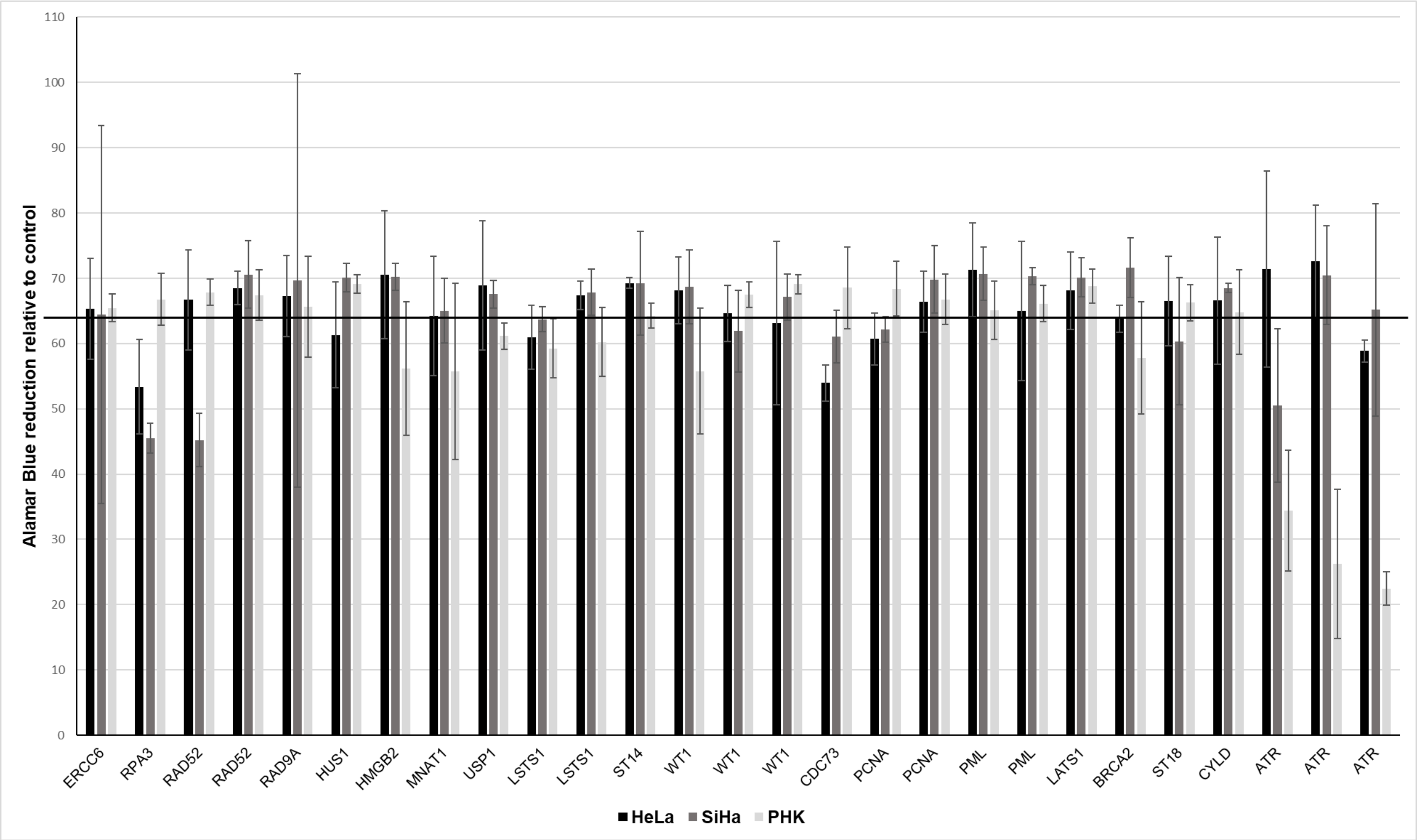

Figure S2– . Screening for genes critical for cervical cancer derived cell lines viability. A lentiviral shRNA library targeting 116 genes involved in DNA damage signaling/repair and tumor suppressors were used to silence specific genes in cervical cancer cell lines and PHK. Cells were seeded in 96 wells plates (2000 cells/well) and infected with lentiviral particles expressing specific shRNAs after 24 hours. Alamar Blue was added (10 µL/well) after 72 hours and its reduction was determined after four hours by absorbance measuring at 570 and 600 nm. Viability/proliferation inhibition values are presented as relative to those observed in control cells (each parental cell line transduced with scramble shRNA). The genes presented in the graph were not selected for further analysis in this study since their silencing, besides reducing SiHa and HeLa cells proliferation, also impacted PHK proliferation to a similar extent.

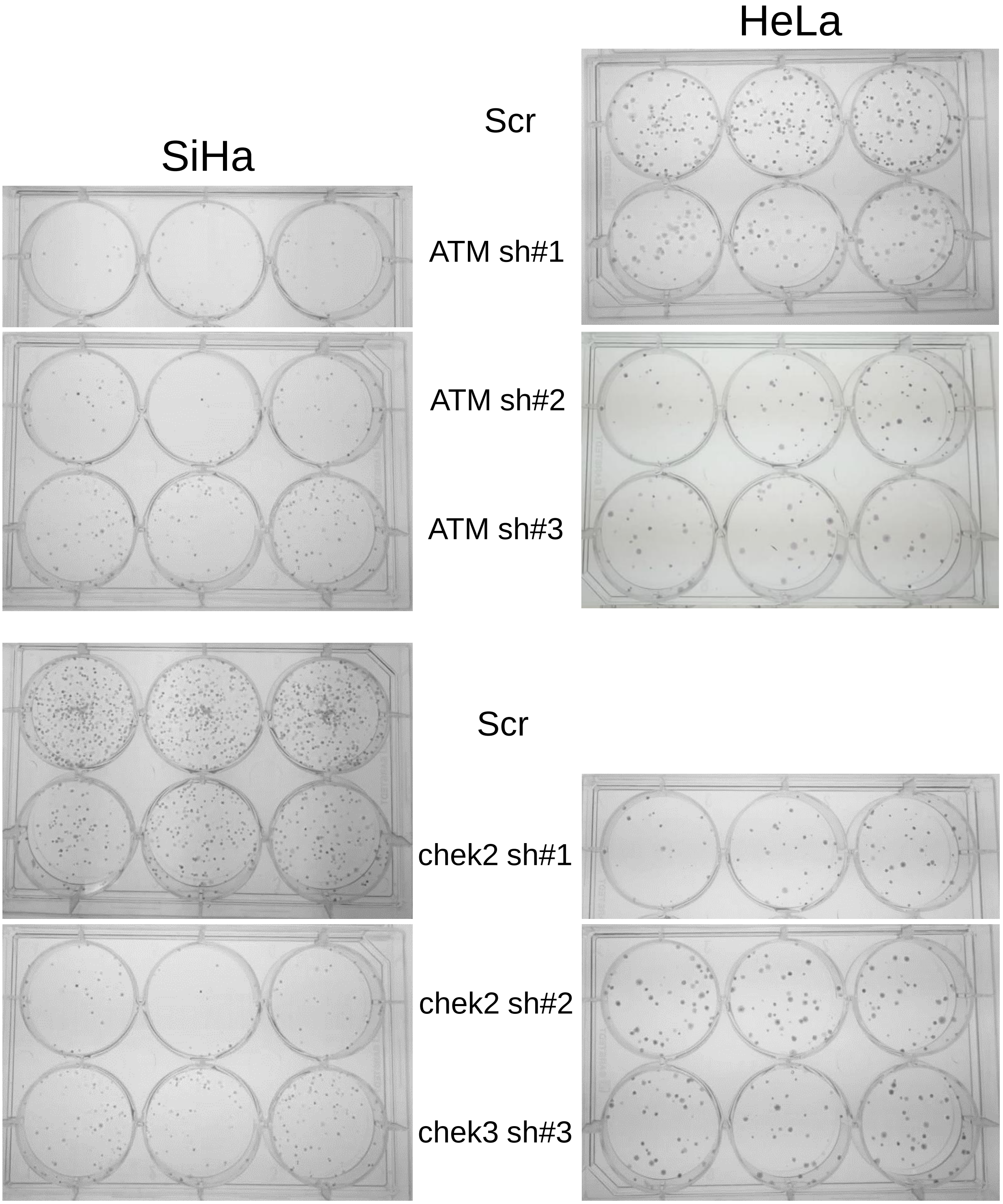

Figure S3– Effect of ATM and chek2 silencing on the clonogenic potential of cervical cancer-derived cell lines. Lentiviral vectors expressing specific shRNA (MISSION® DNA Repair Pathway) that targets ATM and chek2 genes were used to silence specific genes in cervical cancer cell lines HeLa and SiHa. Transduced cells were seeded in 6 wells plates (100 cells/well). After two weeks in culture, clonogenic potential was evaluated. Colonies were fixed with 1% ethanol and stained with 0.5% crystal violet (Fisher, Waltham, MA, USA) in 10% ethanol and counted. Results presented are representative of three independent experiments.

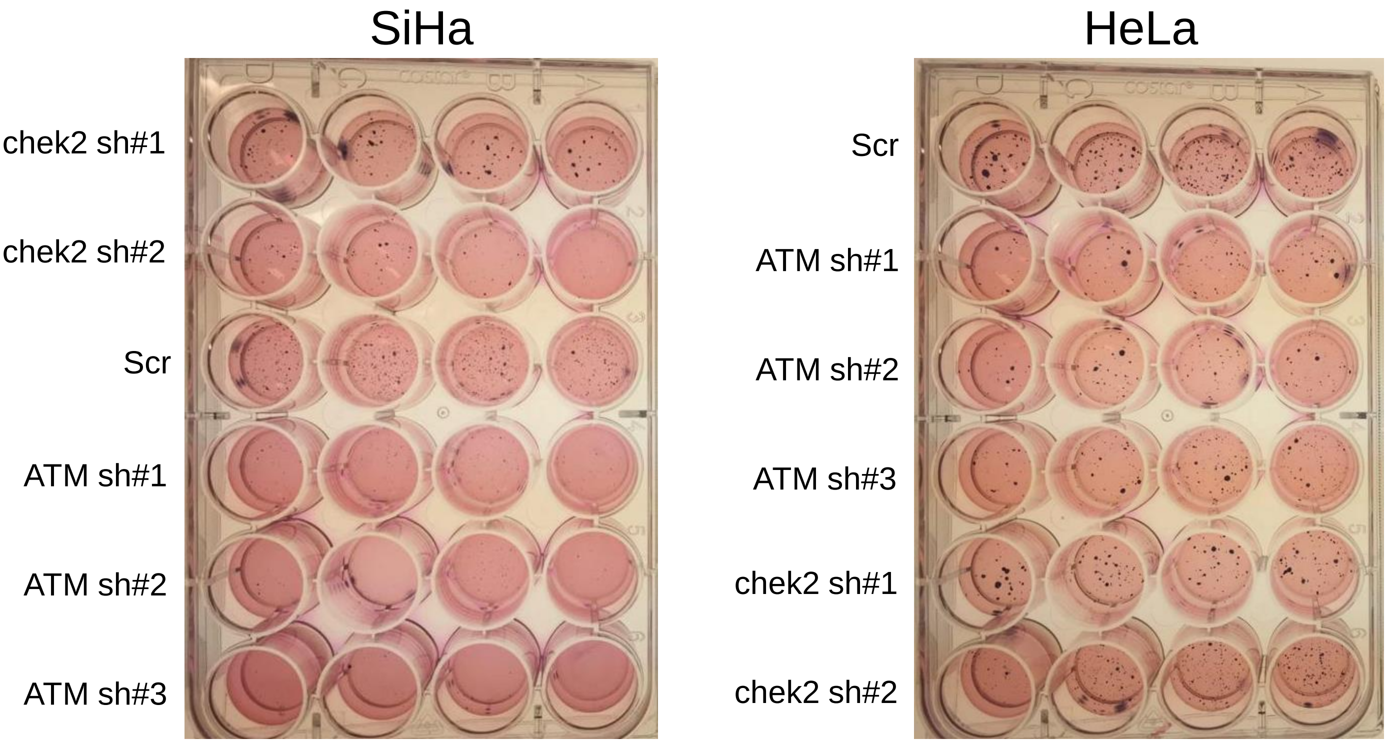

Figure S4 – Effect of ATM and chek2 silencing on the anchorage independent growth potential of cervical cancer-derived cell lines. Lentiviral vectors expressing specific shRNA (MISSION® DNA Repair Pathway) that targets ATM and chek2 genes were used to silence specific genes in cervical cancer cell lines HeLa and SiHa. Transduced cells were resuspended in medium with 0,6% agarose and seeded in 24 wells plates (500 cells/well). After four weeks in culture, colony formation potential was evaluated. Colonies were stained 50 µL of MTT (3-(4,5-dimethylthiazol-2-yl)-2,5-diphenyltetrazolium bromide) and counted. Results presented are representative of three independent experiments.

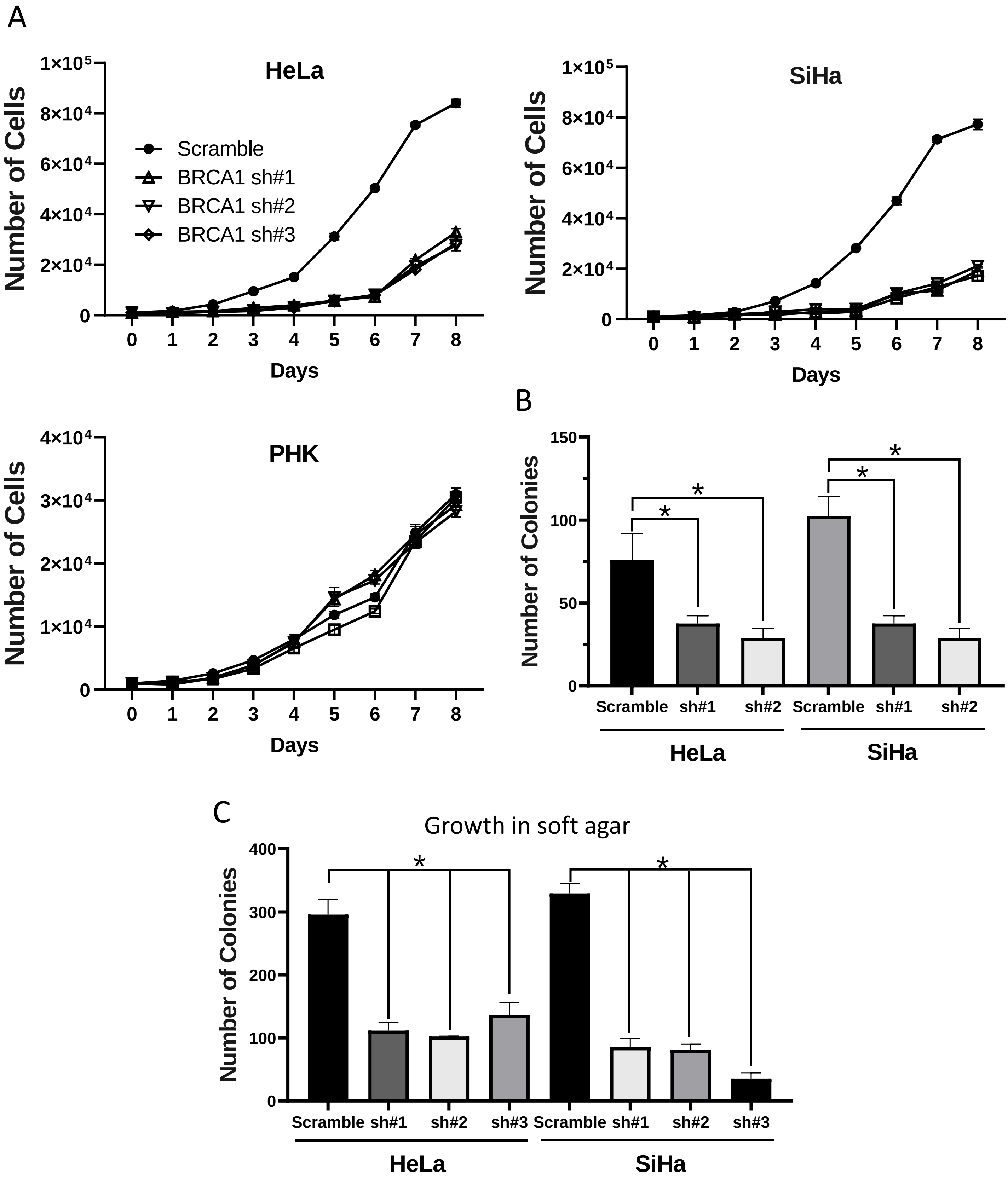
A
HeLa
Number of Cells
1×10⁵
8×10⁴
6×10⁴
4×10⁴
2×10⁴
0
Scramble
BRCA1 sh#1
BRCA1 sh#2
BRCA1 sh#3
0 1 2 3 4 5 6 7 8
Days
SiHa
Number of Cells
Days
PHK
4×10⁴
3×10⁴
2×10⁴
1×10⁴
B
Number of Colonies
150
100
50
*
Scramble
sh#1
sh#2
HeLa
SiHa
C
Growth in soft agar
400
300
200
sh#3

Figure S5 – BRCA1 is essential for the proliferation/viability of HPV-positive cervical cancer-derived cell lines. (A) For growth curves cells were seeded in 24 wells plates (1000 cells/well) and counted daily for 8 days. For clonogenic (B) and anchorage independent growth assays cells were seeded in 6 wells plates (100 cells/well) allowed to grow for two weeks, fixed, stained with crystal violet and counted. For anchorage independent growth assays (C) cells were seeded in 24 wells plates (500 cells/well) and allowed to grow for 30 days. Colonies were stained with MTT and counted. Results are representative of three independent experiments (Student t-test $p<0,05$ for all comparisons between control and treated cells).

Abjaude et al.,

Figure S6

A)

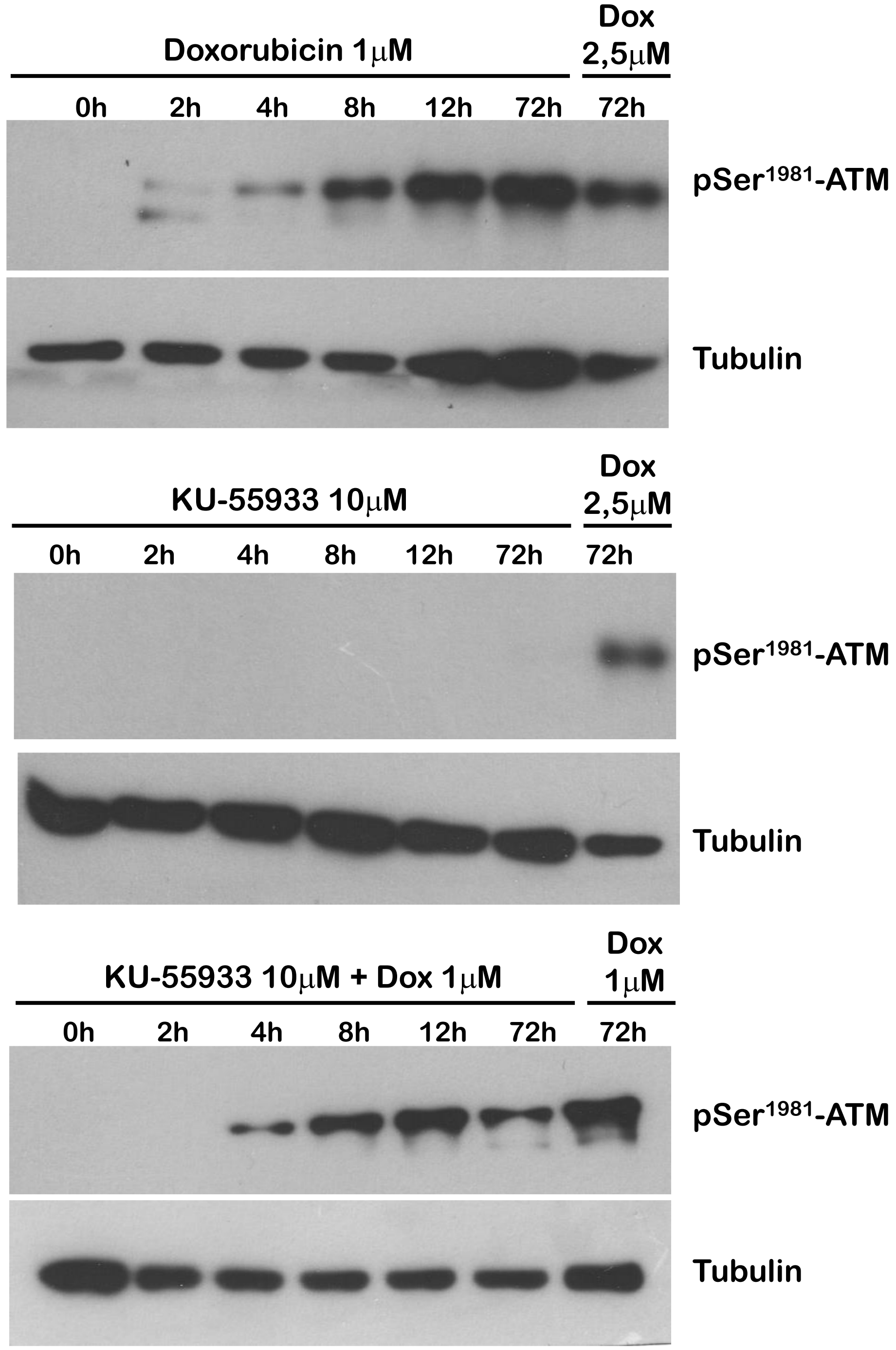

B)

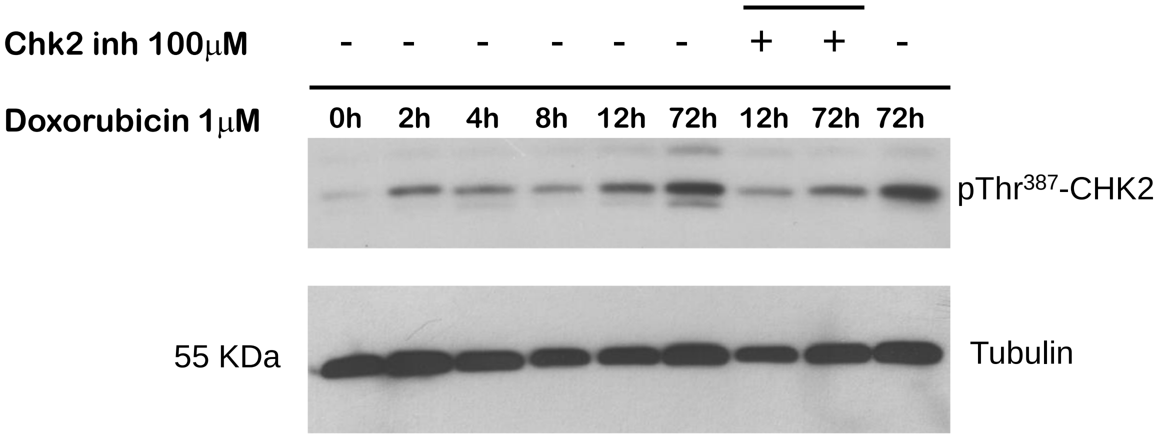

Figure S6 – Effect of ATM inhibitor KU-55933 on ATM activation and of CHK2 inhibitor on CHK2 activation. A) HeLa cells ($3x10^5$ cells/well) were seeded in 6-well plates. After 24h they were treated with doxorubicin (1 or 2,5 μM) and/or the ATM specific inhibitor KU-55933 (10 μM) for 2h, 4h, 8h, 12h and 72h. Thirty μg of total protein extracts loaded in a 4-12% gradient acrylamide gel, separated by electrophoresis and blotted into PVDF membranes. ATM phosphorylation was determined using an anti-phospho S1981 ATM antibody (ab81292). B) HeLa cells ($3x10^5$ cells/well) were seeded in 6-well plates. After 24h they were treated with doxorubicin (1 μM) and/or the CHK2 specific inhibitor 2-(4-(4-Chlorophenoxy)phenyl)-1H-benzimidazole-5-carboxamide hydrate (CHK2 Inhibitor II hydrate) (100 μM) for 2h, 4h, 8h, 12h and 72h. Thirty μg of total protein extracts loaded in a 10% acrylamide gel, separated by electrophoresis and blotted into PVDF membranes. CHK2 phosphorylation was determined using an anti-phospho T387 CHK2 polyclonal antibody (ab55319).

Abjaude et al.,

Figure S7

A)

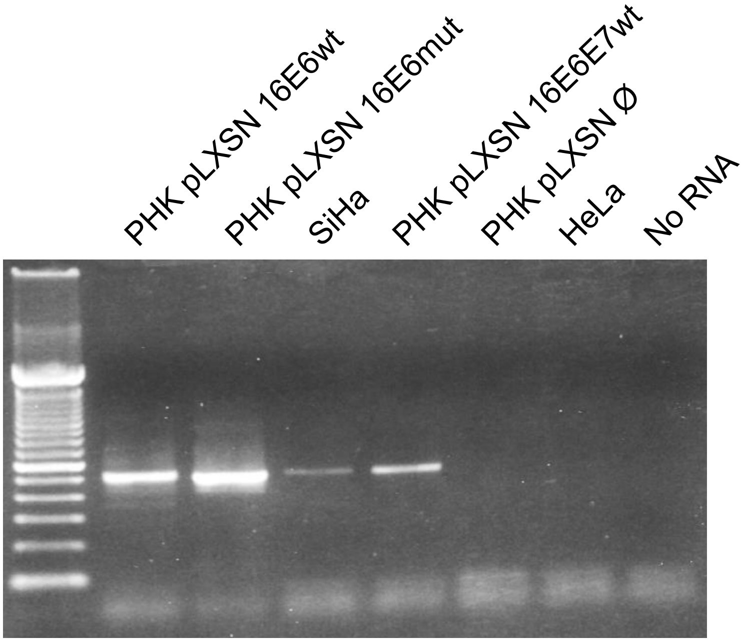

B)

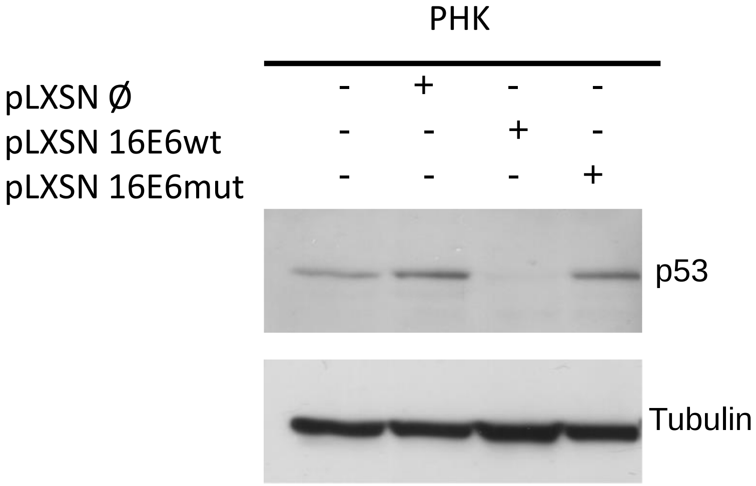

Abjaude et al., Figure S7

C)

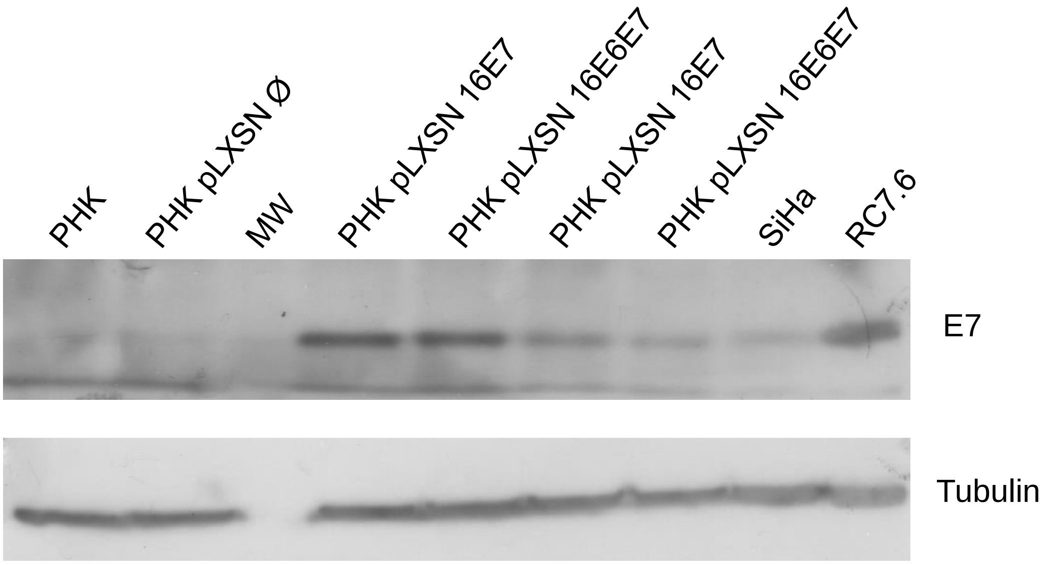

Figure S7 – Confirmation HPV oncogenes expression. PHK were transduced with pLXSN retroviral vectors expressing wild type HPV-16 E6 and/or E7 or a retroviral vector expressing $E6^{8S9A10T}$ (E6mut), unable to bind p53 . A) E6 expression was confirmed by RT-PCR using specific primers (Forward: ATGTTTCAGGACCCACAGG; Reverse: TTACAGCTGGGTTTCTC). B) E6 activity was confirmed by determining p53 degradation by western blot. Thirty μg of total protein extracts were loaded in 10% acrylamide gel, separated by electrophoresis and blotted into PVDF membranes. p53 levels were determined using an specific antibody (anti-p53 (DO-1) sc-126 [Santa Cruz Biotechnology]). C) E7 expression was confirmed by western blot. Thirty μg of total protein extracts were loaded in 12% acrylamide gel, separated by electrophoresis and blotted into PVDF membranes. E7 levels were determined using an specific antibody (Thermo Fisher anti-HPV16 E7 # 28-0006). RC7.6 (Human colorectal carcinoma cells [RKO] transuced with pCMVneo HPV16 E7).

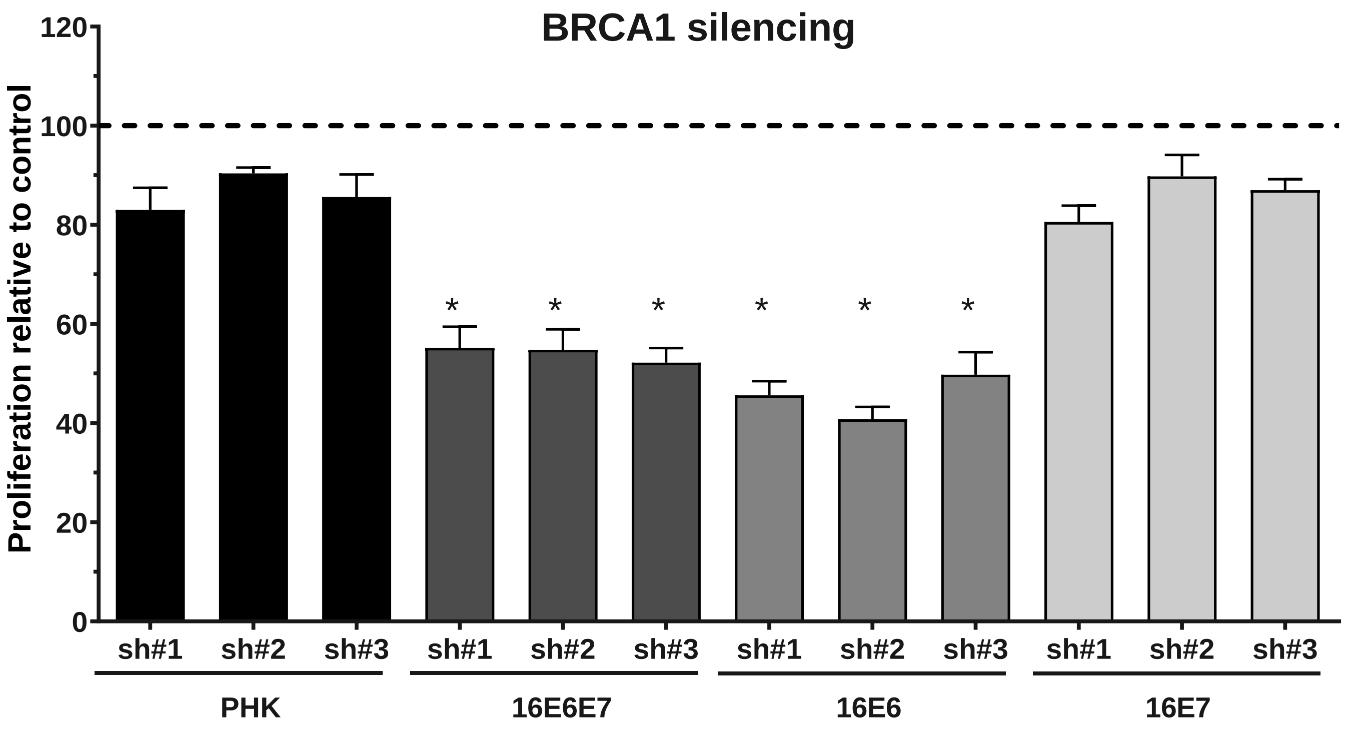

Figure S8 – HPV16 E6 ability to induce p53 degradation is critical to sensitize primary human keratinocytes to BRCA1 silencing. PHK were transduced with retroviral vectors expressing wild type HPV-16 E6 and/or E7. The expression of (A) ATM and (B) BRCA1 was silenced using specific shRNAs. Cells were seeded in 96 wells plates (5.000 cells/well) and after 72 hours their viability was assessed by Alamar Blue reduction. Results presented are representative of three independent experiments (Student t-test p<0,05 for all comparisons between control and treated cells).

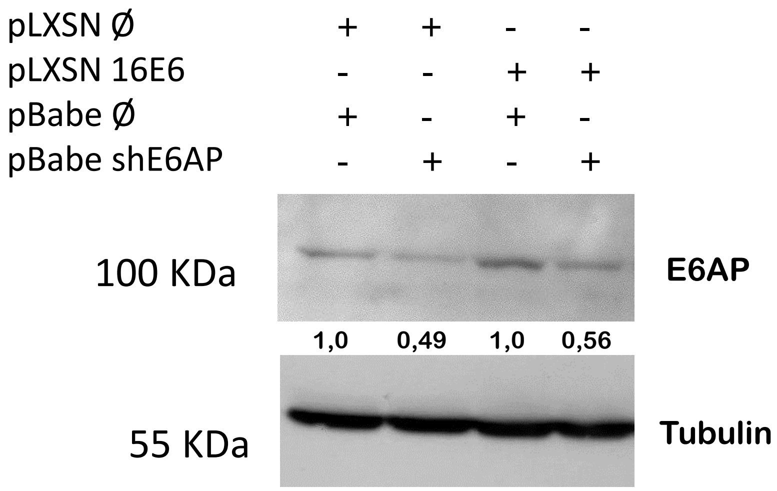

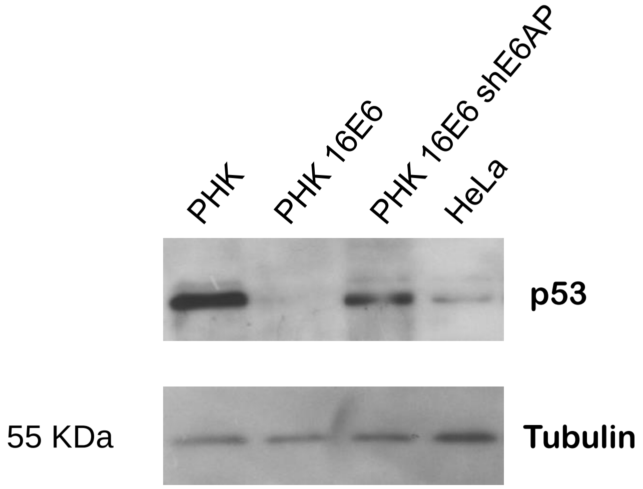

Figure S9 – **Confirmation of E6-AP silencing and p53 induction.** PHK were transduced with retroviral vectors expressing wild type HPV-16 E6 and/or an shRNA targeting E6-AP. Thirty μg of total protein extracts were loaded in 10% acrylamide gel, separated by electrophoresis and blotted into PVDF membranes. E6AP and p53 levels were determined using specific antibodies (ab126765 [Abcam] and anti-p53 (DO-1) sc-126 [Santa Cruz Biotechnology]).
